# Disposition to pediatric intensive care unit post supraglottoplasty repair: a systematic review

**DOI:** 10.1186/s40463-023-00622-z

**Published:** 2023-04-27

**Authors:** Esther ShinHyun Kang, Sena Turkdogan, Jeffrey C. Yeung

**Affiliations:** 1 Faculty of Medicine, McGill University, Montreal, Canada; 2 Department of Anesthesia, McGill University Health Centre, Montreal, Canada; 3 Department of Otolaryngology - Head and Neck Surgery, McGill University Health Centre, Montreal, Canada; 4 Department of Pediatric Surgery, Montreal Children’s Hospital, 1001 Decarie Blvd, Montreal, QC H4A 3J1 Canada

**Keywords:** Supraglottoplasty, Pediatric intensive care unit, Post-operative disposition, Quality Improvement

## Abstract

**Background:**

Patients undergoing supraglottoplasty are often routinely admitted post-operatively to the pediatric intensive care unit (PICU) due to rare but potentially fatal complications such as airway compromise. A systematic review was performed to determine the rate of post-operative PICU-level respiratory support required by pediatric patients following supraglottoplasty, to identify risk factors for patients who may benefit from post-operative PICU admission and limit unnecessary use of intensivist resources.

**Review methods:**

Key search terms ‘supraglottoplasty’ OR ‘supraglottoplasties’ were queried on three databases: CINHAL, Medline and Embase. Inclusion criteria were pediatric patients under 18 years of age who underwent a supraglottoplasty procedure with either an admission to PICU or requirement for PICU-level respiratory support. Risk of bias was assessed by two independent reviewers using QUADAS-2. Findings were critically appraised by three independent reviewers and pooled proportions of criteria meeting PICU admission were calculated for meta-analysis.

**Results:**

Nine studies met inclusion criteria, totaling 922 patients. Age at time of surgery ranged from 19 days to 15.7 years with mean age of 5.65 months. A weighted pooled estimate suggested that 19% (95% CI 14–24%) of patients who underwent supraglottoplasty required PICU-admission. The included studies revealed several patient and surgical factors have been linked to postoperative respiratory issues requiring PICU admission, including: neurological disease, perioperative oxygen saturation < 95%, prolonged surgical time and age < 2 months.

**Conclusions:**

This study found that the majority of supraglottoplasty patients do not require significant postoperative respiratory support and suggests that routine PICU admission of these patients may be avoided by careful patient selection. Given the wide heterogeneity of outcome measures, further studies are needed to determine the ideal PICU admission criteria following supraglottoplasty.

**Graphical Abstract:**

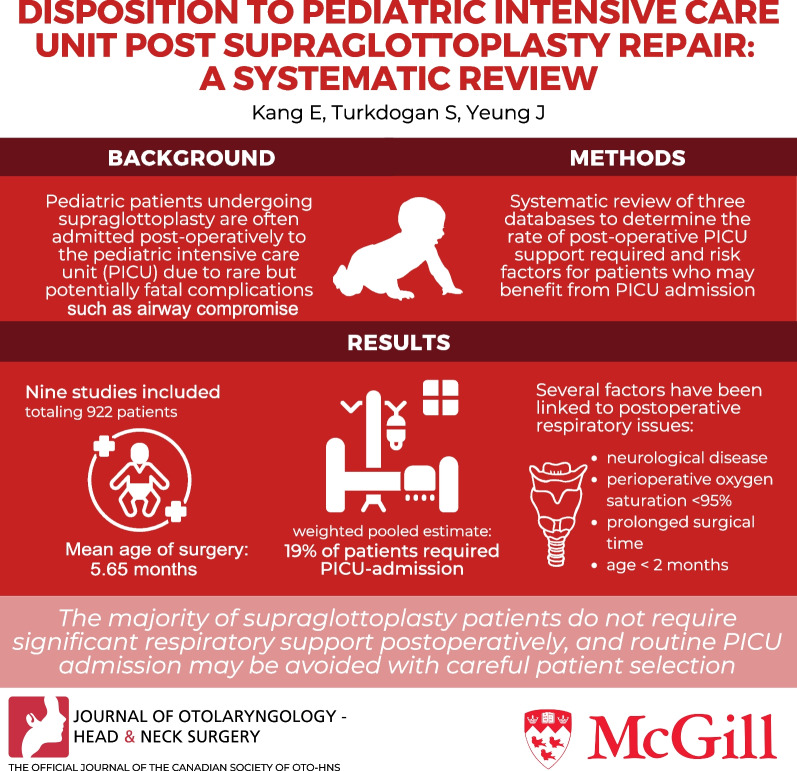

## Introduction

Stridor is a common chief complaint of patients seen in pediatric otolaryngology. While there are many etiologies of stridor in infancy, one of the most common is laryngomalacia [1]. Laryngomalacia is often benign and self-resolving, but when severe, can lead to failure to thrive, hypoxemia or obstructive sleep apnea (OSA), respiratory distress and pectus excavatum [2]. While no definitive treatment algorithm exists, it is these sequelae of laryngomalacia that will often lead the treating team to offer definitive management, namely, supraglottoplasty.

In the long term, overall outcomes of supraglottoplasty are favorable, and those who do eventually require revision surgery are often patients with identifiable risk factors such as congenital anomalies or other comorbidities [2–7]. Critical post-operative airway complications following supraglottoplasty typically occur in the early post-operative course, and include airway edema and aspiration [2, 3]. For this reason, some surgeons advocate for routine intubation post-operatively and/or routine admission to the pediatric intensive care unit (PICU). However, the proportion of patients who in fact require PICU-level respiratory support following supraglottoplasty, such as high-flow oxygen, non-invasive positive pressure ventilation and/or intubation is variable [3, 4]. Often, the reason for PICU admission is mainly to provide continuous cardiac and respiratory monitoring, the smaller patient-to-nursing ratio, and the resources for elevated interventions if this becomes necessary. For this reason, many hospitals have focused on developing “step-down” or “intermediate care” units [8].

The extensive use of PICU beds creates an undue burden to the healthcare system both financially and medically. PICU beds consume a substantial amount of hospital and personnel resources. In fact, it is globally one of the most expensive hospital admissions ranging from $2078 to $5857 USD per day, as much as a four-fold increase ($459.94–$1771.85 USD) compared to a surgical ward [9–11]. This potentially unnecessary increased cost-of-stay has repercussions for quality-based funding and renumeration of supraglottoplasty procedures.

The objective of the current study was to systematically review the available peer-reviewed literature, to determine a pooled-estimate of the rate of post-operative PICU-level respiratory support required by pediatric patients following supraglottoplasty and to identify risk factors for patients who may benefit from post-operative PICU admission. We hypothesize that routine admission to PICU may be over-utilized in the supraglottoplasty population, and that PICU-level respiratory support post-supraglottoplasty should be reserved for select populations.

## Methods

### Review questions

This review sought to establish the best practice for determining patient disposition to the PICU post-operatively following supraglottoplasty based on the available literature. These specific questions were addressed:

- What is the incidence of routine PICU admission?
- What is the incidence of PICU-level airway support required in pediatric patients undergoing supraglottoplasty?
- What are the associated clinical characteristics and risk factors in patients that increase the need for post-operative PICU-level respiratory support?

### Search design

The inclusion criteria comprised of all studies that involved human subjects between ages of 0–18 years who had undergone a supraglottoplasty procedure. Studies of interest included those that discussed admission to the PICU and PICU-level respiratory support, which was defined as any of the following: administration of high-flow oxygen, non-invasive positive pressure ventilation (NIPPV), nebulized racemic epinephrine, and/or intubation [12]. All types of studies were included in the search with the addition of conference abstracts, ongoing clinical trials and book chapters. Studies were excluded if they were not written in English or included an age range older than 18 years old.

The primary outcome of interest was the incidence of administration of PICU-level respiratory interventions including prolonged intubation, re-intubation, nebulized epinephrine, positive pressure ventilation and oxygen therapy delivering more than 4L via nasal prongs. Secondary outcomes included clinical characteristics identified in patients requiring PICU-level respiratory support. General demographic information was also collected. The extracted data were synthesized on a separate table on a word document.

Our key search term was ‘supraglottoplasty’ OR ‘supraglottoplasties’ identified in the title, abstract or subject descriptors. Only two key search terms were used as the focus of the review is on the disposition post-supraglottoplasty, unrelated to the condition that required to patient to undergo the procedure. Several combinations of key words including ‘supraglottitis’ and ‘laryngomalacia’ were used, however, these only further diluted our search results as laryngomalacia and supraglottitis patients treated medically and/or expectantly were out of the scope of this review. Articles were searched in three different databases: CINAHL, Medline/Pubmed, Embase.

Search results were then uploaded onto EndNote (Clarivate Analytics, Philadelphia, PA, USA). Titles and abstracts were screened by two independent reviewers (ESK and JY) and discordant abstracts and articles were reviewed by a third reviewer (ST) before a consensus was obtained amongst all authors. After full-text review was performed, data was extracted from the search results by an independent reviewer (ST) in a standardized fashion. Reporting of the systematic review was performed following the PRISMA guidelines.

### Risk-of-bias assessment and quality assessment

The risk-of-bias assessment was performed independently by two reviewers (ESK and ST) using the QUADAS-2 tool by the University of Bristol. This tool assessed the risks of bias in the following domains: patient selection, index test, reference standard, and flow and timing as well as applicability concerns in the same domains.

### Data extraction and statistical analysis

Data was extracted from fully reported articles and any discrepancies were resolved by consensus between the three reviewers. Risk factors for PICU admission were categorized, the data was assessed for heterogeneity, and pooled proportions of criteria meeting PICU admission were calculated for meta-analysis. Calculations were performed using the statistical software package STATA-13 (STATA Corporation, College Station, TX, USA).

## Results

### Description of studies

**Fig. 1:**
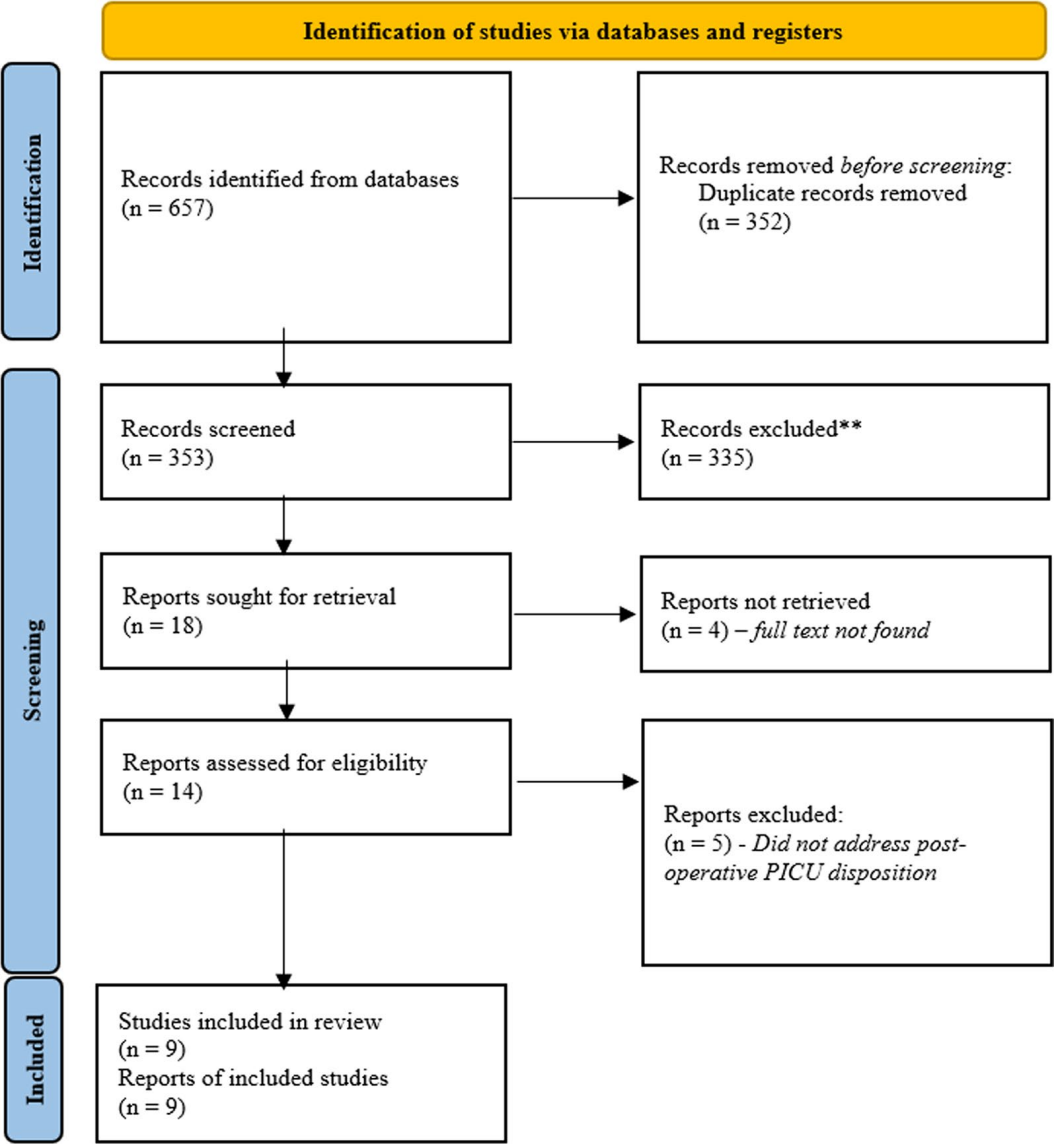
PRISMA flowchart of study cohort

A total of 657 entries were identified from the primary databases (Medline/Pubmed: 223, EMBASE: 344, CINAHL: 90). The list was de-duplicated resulting a total of 352 unique search findings (Medline/Pubmed: 82, EMBASE: 223, CINAHL: 47). Of the 352 findings, 18 were selected by two independent reviewers for full-text assessment. Four were excluded as the selected abstracts did not have a published full-text manuscript and 5 were excluded because they did not address post-operative ICU disposition. Finally, nine were included for the final review (Fig. 1, Table 1) [9, 10, 13–19].

**Table 1:** Characteristics of included studies—NR: Not recorded

First author	Date	Country	Design	N	Age, mean (range)	Sex (M/F)	ICU Admissions, proportion (%)	ICU Criteria Met, proportion (%)	Intubation/re-intubation, proportion (%)
Albergotti	2015	USA	Retrospective case series	223	0.59 (0–15.7) years	103/120	205/223 (91.9%)	25/223 (11.2%)	9/223 (4.0%)
Chan	2017	UK	Retrospective case series	42	4.9 (0.2–39) months	19/23	42 (100%)	12/42 (28.6%)	5/42 (11.9%)
Chen	2019	USA	Retrospective case series	98	3.4 (IQR 2.0–6.3) months	62/36	NR	40/98 (40.8%)	9/98 (9.2%)
Cooper	2017	Canada	Retrospective cohort study	141	Routine group: 1.16 ± 2.70 years	25/10	25/35 (71.0%)	24/141 (17.0%)	24/141 (17.0%)
					Selective group: 1.52 ± 2.11 years	63/43	28/106 (25.0%)		
Fordham	2013	USA	Retrospective case series	65	3 months (NR)	-	1/65 (1.5%)	1/65 (1.5%)	0
Lee	2007	Taiwan	Retrospective case series	138	6.97 (0–54) months	79/59	138/138 (100%)	NR	NR
Schroeder	2009	USA	Retrospective case series	52	8 months (NR)	-	52/52 (100%)	33/52 (63.0%)	17/52 (32.7%)
Yeung	2020	USA	Retrospective cohort study	155	8 (IQR 3–22) months	96/59	155/155 (100%)	25/155 (16.1%)	7/155 (4.5%)
Zainal	2011	Malaysia	Retrospective case series	8	15.6 months (NR)	7/1	NR	6/8 (75.0%)	5/8 (62.5%)

The nine studies meeting inclusion criteria totaled 922 patients. Eight of nine studies were retrospective case series, and one was a retrospective cohort study. Five studies were conducted in USA, and one in each of the following countries: Canada, Malaysia, United Kingdom. Patient age at time of surgery ranged from 19 days to 15.7 years with mean age of 5.65 months.

### Risk of bias assessment

**Fig. 2:**
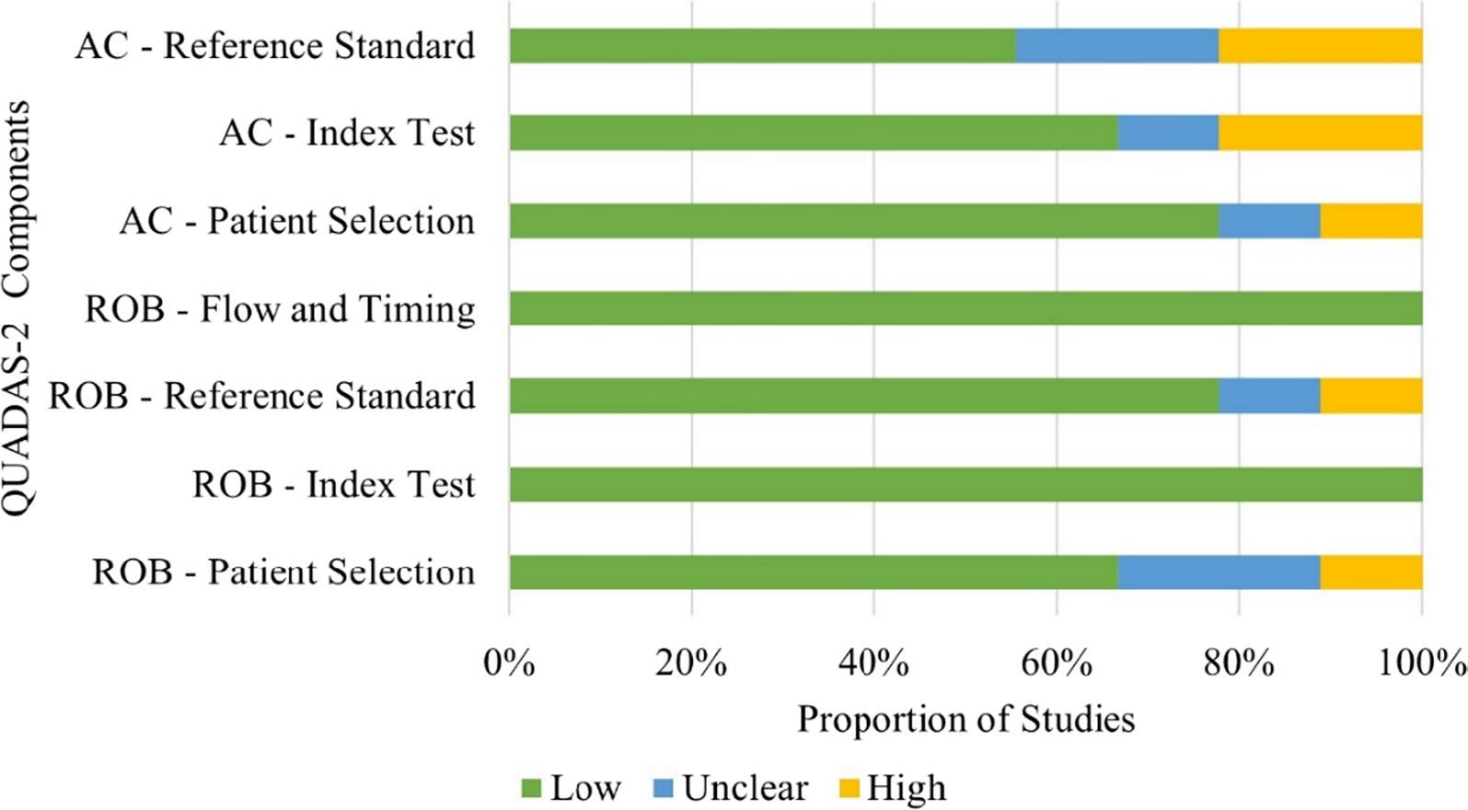
Graphical display of QUADAS-2 Quality Assessment of Included Studies. *AC* applicability concerns; *ROB* risk of bias

Five studies were judged to be low risk of bias in all domains. Three studies were found to have unclear bias, specifically in their patient selection secondary to extensive exclusion criteria. One of the three studies also had an unclear bias in reference standard. One study was judged to have a high risk of selection and reference standard bias. Only four studies had low applicability concerns. Two studies were judged to have unclear concerns and three had high applicability concerns (Fig. 2).

### Outcome measures

#### PICU admissions and true requirements

**Fig. 3:**
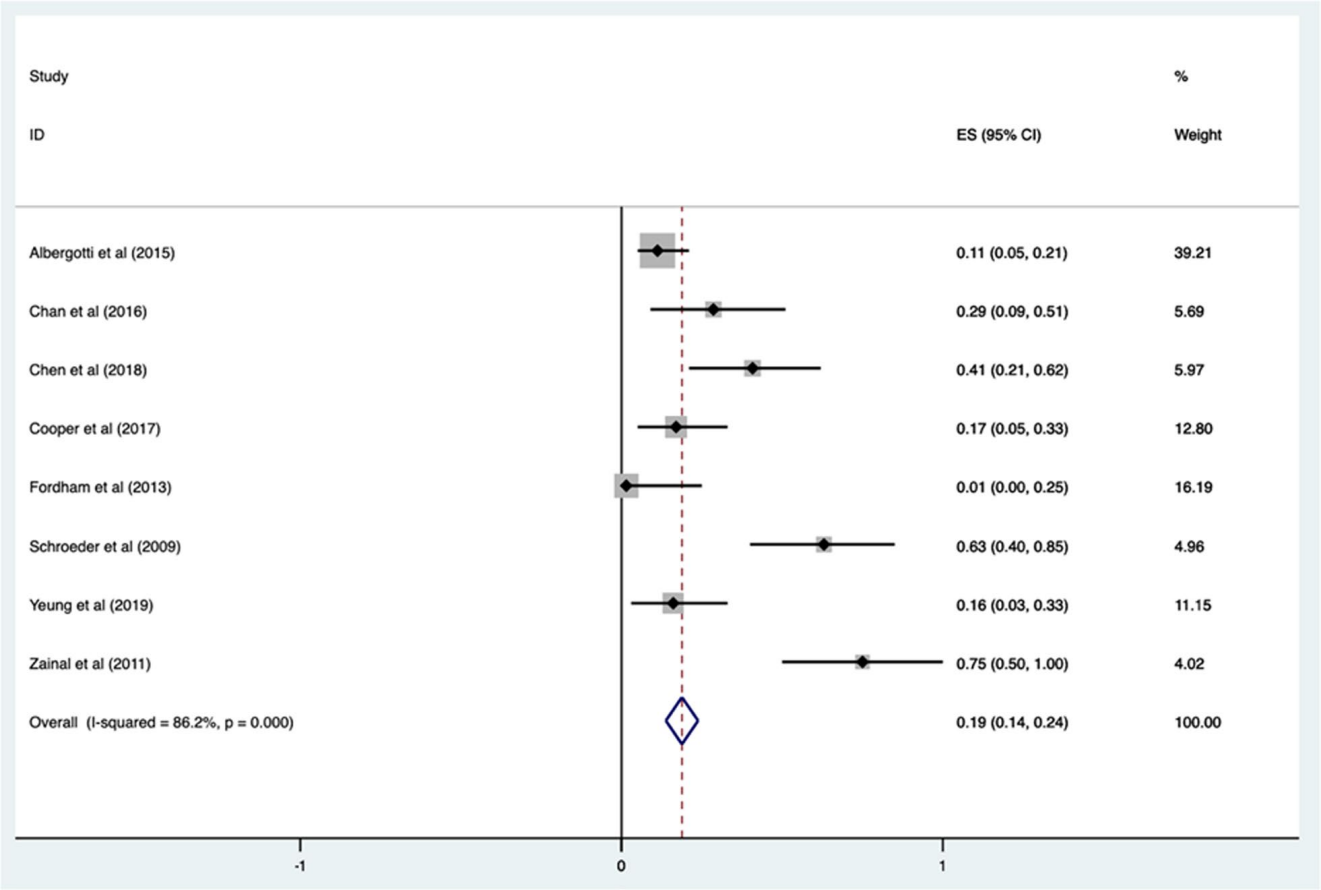
Number of patients meeting criteria for admission in a pooled fixed-effect analysis

In seven of the nine studies where PICU admissions were assessed, a total of 626/718 (87.1%) patients were routinely admitted to the PICU post-supraglottoplasty. When a weighted pooled proportion was applied, only 19% (95% CI 14–24%) of patients met PICU admission criteria (Fig. 3). Heterogeneity was 86.2%. In the two other studies where patients had an option for admission on a step-down unit [9, 10], 43/204 (21.1%) of patients were selected for admission to the PICU. Based on our admission criteria which included intubation/re-intubation requirement, use of nebulized racemic epinephrine, prolonged or high-flow O_2_ requirement post-operatively, and NIPPV, only 166/784 (21.2%) patients required admission to PICU post-operatively (Table 2).

**Table 2:** Possible risk factors associated with PICU-level intervention

Patient factors	Surgical factors
Underlying neurological disorders Underlying pulmonary disorders and OSA Non-white race Younger age Perioperative oxygen saturation < 95%	CO_2_ laser Microscope Prolonged surgical time > 30 min

Given that many non-invasive respiratory support measures can theoretically be carried out in a step-down units, depending on institutions, the need for PICU admission was re-evaluated with intubation for mechanical ventilation as the sole criteria. Using this criterion, only 76/784 (9.7%) of patients required prolonged intubation (26/784; 3.3%) or re-intubation (50/784; 6.4%) post-supraglottoplasty warranting admission to the PICU.

#### Significant risk factors

The nine studies analyzed various risk factors to predict association with post-supraglottoplasty PICU requirements. Three studies totaling 305 patients [14, 17, 18] identified underlying neurological and neuromuscular disease as a risk factor for PICU-level respiratory support. Two studies totaling 265 patients [10, 13] identified pre-operative O_2_ requirements for SpO_2_ > 94% or intra-operative PaO_2_ < 90% as a risk for post-operative PICU-level respiratory support. Four studies totaling 518 patients [10, 13, 14, 18] observed that the mean age was younger in the PICU group, though, this association was not statistically significant. A single study also identified non-white race, bilateral trimming of aryepiglottic folds, prolonged surgical time > 30 min and the use of microscopes intraoperatively as significant risk factors for PICU admission [13]. The same study also identified preoperative diagnosis of gastric esophageal reflux (GERD) to be protective of post-operative PICU needs. Other significant risk factors identified in single studies include underlying pulmonary disease and obstructive sleep apnea [14], the use of carbon dioxide (CO_2_) laser [18], pre-existing laryngeal edema and synchronous airway lesions which are defined as “airway anomalies (other than laryngomalacia) that may contribute to upper airway obstruction, including vocal fold motion impairment, subglottic stenosis (SGS), tracheomalacia, and bronchomalacia” [17].

## Discussion

This systematic review sought to summarize the existing literature regarding early post-operative outcomes of patients undergoing supraglottoplasty with an aim of determining the optimal disposition of these patients. The current body of literature suggests that approximately 80% of patients may not in fact require PICU admission after supraglottoplasty procedure, even though many institutions/surgeons continue to routinely admit these patients to the PICU.

Our results demonstrated that despite routine admission, only about 20% of patients truly required PICU-level respiratory interventions. Certainly, this data suggests that keeping all patients routinely intubated post-supraglottoplasty may not be necessary. Of course, some patients with risk factors suggestive of a more complicated post-operative course may benefit from an extended period of intubation. The objective of this systematic review was in fact to identify such risks. By developing a more robust PICU admission criteria, the number of post-supraglottoplasty PICU admissions may be reduced and along with some of the psychosocial burden placed on patients and their families. Two studies in this review specifically described postoperative admission to “step-down” units as a potential disposition for these patients [9, 14]. These wards are organized to have a smaller patient-to-nurse ratio, close monitoring capability and enhanced respiratory support when compared to normal surgical or medical wards.

In the similar situation of transoral laryngeal cleft repair, the concept of a Pediatric Perioperative Surgical Home (PPSH) model has been described specifically to determine a patient’s necessity for post-operative PICU admission [20]. In this model, the authors proposed pre-operative triage of patients, dividing them into high-acuity populations (significant medical comorbidities or craniofacial abnormalities) who need ICU care versus low-acuity patients who could be admitted to a “step-down” unit. At the institution of study, selective post-operative admission criteria significantly reduced ICU utilization in this patient population (143 ICU bed days pre-PPSH). This intermediate care setting may be the ideal setting for post-supraglottoplasty disposition.

The risk factors associated with PICU-level respiratory support that have been identified to-date include (1) patient factors, namely underlying neurological or pulmonary disorders, non-white race, and pre-operative/intra-operative oxygen requirement and (2) surgical factors, namely the use of CO_2_ laser, the use of the operating microscope, and prolonged surgical time > 30 min. Unfortunately, the heterogeneity of the data in the current study limited any further meta-analysis, and pooled estimates of individual patient and surgical risk factors could not be determined.

### Patient factors

The association between neurological comorbidities and worse prognosis and post-operative outcomes has been long understood and perhaps unsurprising [21]. These patients have been found to have higher rates of surgical failure following supraglottoplasty, persistent OSA, and need for revision surgery and/or tracheostomy [22, 23]. This suggests that neuromuscular tone is a significant factor in disease course in neurologically compromised patients with concomitant laryngomalacia [22]. Regarding race as a risk factor, very few of the included entries studied non-white race as a risk factor for post-operative PICU intervention. Yet interestingly, one study investigating ethnic differences in laryngomalacia showed that the condition is prevalent in African American children and suggested that the significant anatomical variations between races that may render the results of Caucasian-dominant literature less applicable to a diverse patient population [24].

Younger age was observed in patients admitted to the PICU in the studies included in our review, but not found to be statistically significant. However, in two studies specifically investigating young patients undergoing supraglottoplasty (less than 2 months), age was found to be associated with a more difficult post-operative course, including prolonged intubation, which corresponds with the observations made in our studies [23, 25].

While there are no specific studies evaluating the impact of perioperative oxygen saturation on patients’ post-supraglottoplasty course of recovery, several studies identified perioperative oxygen saturation < 95% in general as a risk factor for early post-operative hypoxemia, which may explain the results observed herein [26, 27]. Similarly, prolonged surgical time in general was found to be associated with unplanned admission to the PICU [28]. It may be expected that these are especially true for patients who have undergone airway surgery.

Pre-existing laryngeal edema and synchronous airway lesions, specifically, subglottic stenosis > 35% were also noted by one study to be significant risk factors for post-operative PICU requirement [17]. Post-operative airway edema and supraglottoic stenosis are some of the feared complications of surpaglottoplasty and common arguments made for routine PICU admissions [2]. Thus, it is a reasonable association that underlying laryngeal edema and airway lesions may cause post-operative airway obstruction requiring more invasive interventions. Along the same lines, pre-existing diagnosis of GERD was reported by one study to be a protective factor to post-operative PICU requirements. The authors explained that this was likely due to its relationship with greater use of antireflux medication [13]. Although antireflux medication may not treat the underlying anatomical defect or reduce the frequency of supraglottoplasty [29], but it is known to reduce laryngeal edema which may explain the results of its protective factor [30].

### Surgical factors

Interpreting data on the intraoperative surgical factors is challenging as an association identified retrospectively does not account for an individual surgeon’s skill, experience, and technique. In this systematic review, surgical factors that have been associated with PICU-admission included use of the operating microscope, bilateral trimming of aryepiglottic folds, use of CO_2_ laser, and prolonged surgical duration. The authors who reported the first two risk factors described that both the use of the microscope and bilateral trimming of folds were significant on univariate analysis but not significant on multivariate analysis. Furthermore, they explained that rather than being independent risk factors, they were considered to be markers of severe laryngomalacia or complicated surgical procedure which likely prolonged surgical duration—an independent risk factor. The two studies reporting data on the use of the CO_2_ laser had conflicting results, with one study not identifying a positive association and a second in disagreement [18]. Much debate has been brought up in literature regarding the use of CO_2_ laser in laryngeal surgery; opponents have pointed out risk of post-operative aspiration [31], airway fire and thermal damage [32], while its proponents will speak to its precision and hemostatic ability, and its lack of collateral thermal damages [33]. Furthermore, a surgeon’s decision to use the CO_2_ laser may itself be the result of another factor, such as the ease of intra-operative visualization or the ability to ventilate without use of supplemental oxygen. While an association between each of the above risks and respiratory complications following supraglottoplasty has been described reported previously, it is important to note that (a) the finding has not been consistent, and (b) methodologically speaking, no study to-date has been inadequate to confirm ay causal link between use of the CO_2_ laser and respiratory compromise.

Supraglottoplasty is a relatively safe surgical procedure with reported success rates of 70–100% [7], and low risk of severe long-term complications [2, 3]. One must then consider that the burden of a PICU admission on patients and caregivers, and the healthcare system may be quite significant. There is no doubt that a tenuous or obstructed airway must be secured, but it is important to note that neither mechanical ventilation nor NIPPV are benign interventions and should be used judiciously. Delayed extubation or prolonged intubation in neonates and children can cause ventilation associated pneumonia, airway stenosis, and/or opioid withdrawal [34]. Moreover, a recent study identified substantial adverse psychological outcomes associated with PICU admissions in young children [35]. While these results were mostly associated with long-term stay, the psychosocial stress experienced by patients and families from the critical care setting is not negligible even in a short admission. This example was recently amplified and highlighted as visitation hours were restricted during the SARS-COV-2 pandemic. Thus, the decision to intubate a child must be carefully considered and the current data would not support the use of routine post-operative intubation following supraglottoplasty.

There were several limitations to this study. First, all included studies were retrospective and therefore carry an inherent risk of selection bias. Specifically, all studies retrospectively reported the subjective clinical decisions made by the treating team regarding disposition and level of respiratory intervention. The subjectivity of clinical decision-making also applied to indication(s) to proceed with supraglottoplasty. Furthermore, due to the specificity of the study question, only 9 articles ultimately met inclusion criteria, of which only 7 had low to unclear risks of bias and applicability concerns (Table 1).

## Conclusion

This study offers an up-to-date summary of the current literature on postoperative disposition following supraglottoplasty. The data demonstrate that when candidates for supraglottoplasty are carefully selected, routine PICU admission may be avoided in greater than 80% of cases. Our review suggests that underlying neurological or pulmonary disorders, laryngeal edema, synchronal airway lesions, pre-operative oxygen requirement, intraoperative PaO_2_ < 90%, non-white race, prolonged surgical time and the use of CO_2_ laser may be associated with post-operative PICU interventions. Thus, tailoring PICU admissions in a selective fashion could result in a meaningful reduction in intensive care utilization and the psychological burden placed on families and caregivers. Future prospective studies are needed to determine the exact risk profile necessitating PICU admission following supraglottoplasty.

## Data Availability

All data generated or analyzed during this study are included in this published article [and its supplementary information files].
